# Strangulated Spigelian Hernia With Small Bowel Obstruction Requiring Intestinal Resection: An Uncommon Cause of Acute Abdomen

**DOI:** 10.7759/cureus.110416

**Published:** 2026-06-07

**Authors:** Raúl Porras Gutiérrez de Velasco, Florentino Antonio Jimeno Salazar, Daniel Saldaña Merlan

**Affiliations:** 1 Department of General Surgery, Institute for Social Security and Services for State Workers (ISSSTE) General Hospital “Gral. José María Morelos y Pavón”, Mexico City, MEX

**Keywords:** acute abdomen, bowel necrosis, intestinal obstruction, spigelian hernia, strangulated hernia

## Abstract

Spigelian hernia is a rare abdominal wall hernia. It occurs more commonly in women between the fifth and seventh decades of life and is typically located within the Spigelian belt. Because of its interparietal location and small fascial defect, diagnosis may be challenging, and the risk of incarceration and strangulation is high. We present the case of a 64-year-old woman who presented to the emergency department with abdominal pain, nausea, and obstipation. Clinical evaluation was consistent with an acute abdomen secondary to intestinal obstruction. Emergency exploratory laparotomy revealed a Spigelian hernia containing a strangulated segment of terminal ileum with associated bowel necrosis. Segmental small bowel resection with primary anastomosis was performed. The postoperative course was uneventful, with gradual reintroduction of oral intake, and the patient was discharged on postoperative day seven without complications. Spigelian hernia should be considered in the differential diagnosis of patients presenting with acute abdomen and intestinal obstruction without an evident cause. Early diagnosis and prompt surgical intervention are essential to prevent complications such as bowel ischemia, necrosis, and perforation. This case highlights the clinical importance of maintaining a high index of suspicion for this uncommon entity in emergency surgical practice.

## Introduction

Spigelian hernia is a rare type of abdominal wall hernia, accounting for approximately 0.1% to 2% of all cases [1-4]. It occurs more frequently in women between the fifth and seventh decades of life, with a reported female-to-male ratio of approximately 1.4:1 [1].

Approximately 90% of Spigelian hernia cases arise within the so-called “Spigelian belt,” a transverse anatomical zone approximately 6 cm in width located above the interspinous line [5]. This region represents an area of relative fascial weakness, predisposing to hernia formation [6].

A notable clinical feature of Spigelian hernia is the small size of the fascial defect, typically less than 2 cm, which significantly increases the risk of incarceration and strangulation of hernia contents [6]. Consequently, approximately 17% to 25% of cases present as surgical emergencies [1,3,7].

The hernia sac most commonly contains preperitoneal fat, omentum, or small bowel loops [1,4,7]. However, unusual presentations have been reported, including cases of acute appendicitis within the hernia sac, representing a rare and diagnostically challenging scenario [2].

The aim of this report is to describe a case of Spigelian hernia complicated by strangulation of the terminal ileum, highlighting its clinical presentation, diagnostic approach, and surgical management.

## Case presentation

A 64-year-old Mexican Hispanic woman presented to the Emergency Department of Hospital Regional General José María Morelos y Pavón, ISSSTE, Mexico City, Mexico, with a five-day history of abdominal pain associated with nausea without vomiting and obstipation for four days. She had previously been evaluated at another healthcare facility, where she received analgesic treatment with partial symptom relief and was subsequently discharged. Due to persistence of symptoms, she sought further medical attention.

Her medical history was significant for systemic arterial hypertension of 20 years’ duration, treated with felodipine, and type 2 diabetes mellitus for 10 years, managed with metformin. Her surgical history included hysterectomy 20 years prior, uterine curettage, open umbilical hernia repair with prosthetic mesh placement three years earlier, and excision of breast cysts 18 years prior. She also reported a prior blood transfusion due to severe anemia and a history of left wrist fracture.

On physical examination, the patient was hemodynamically unstable (blood pressure: 70/44 mmHg, mean arterial pressure: 53 mmHg, heart rate: 95 beats/minute, respiratory rate: 22 beats/minute), requiring vasopressor support, with findings suggestive of systemic inflammatory response syndrome, including tachypnea, tachycardia, and leukocytosis. The abdomen was distended, predominantly due to adipose tissue, with decreased bowel sounds, muscular guarding, and pain on medium and deep palpation, along with signs of peritoneal irritation.

**Figure 1:**
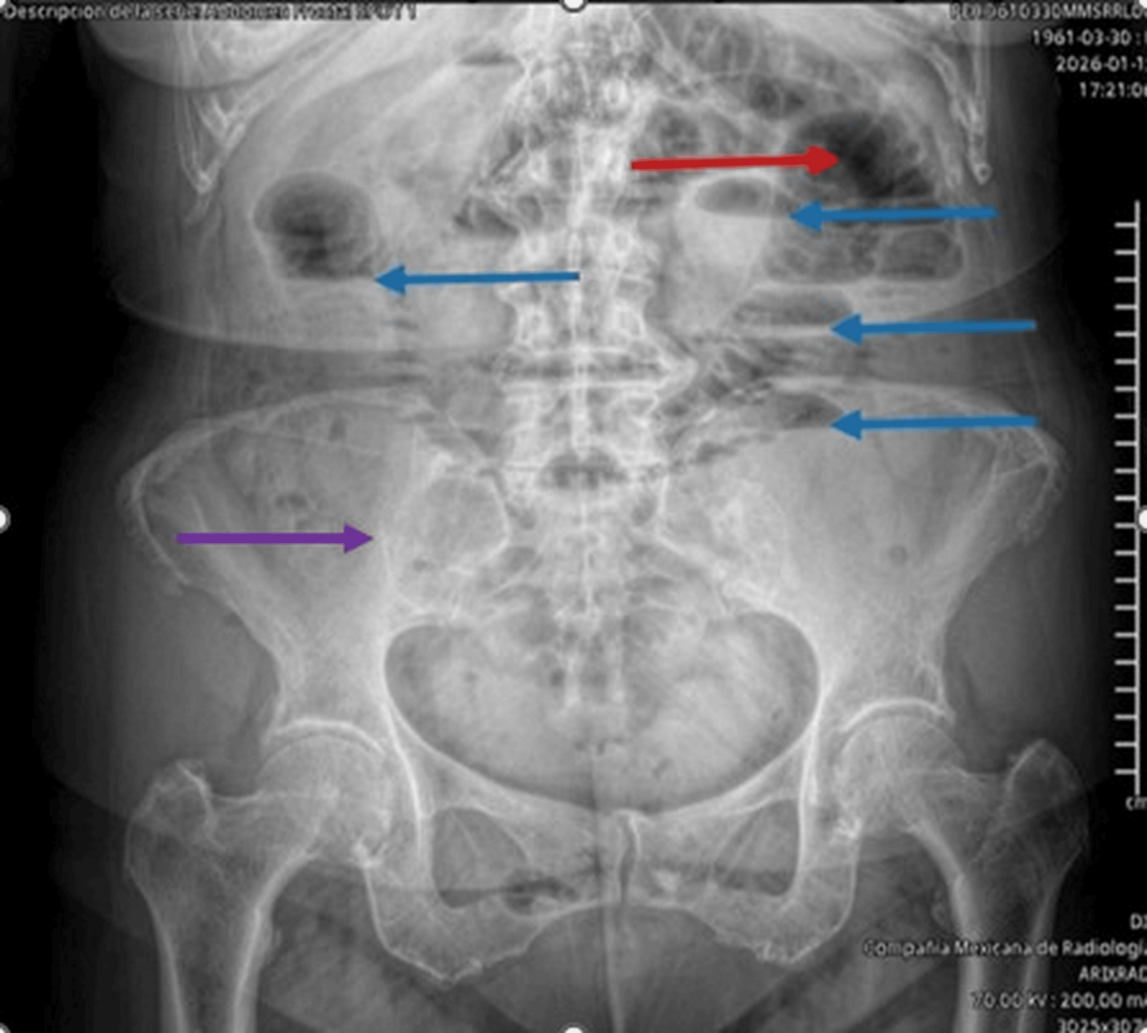
Upright abdominal radiograph showing multiple dilated small bowel loops (red arrow) with air-fluid levels in a step-ladder pattern (blue arrows) and a strangulated bowel loop within a Spigelian hernia (purple arrow), findings suggestive of mechanical small bowel obstruction.

**Figure 2:**
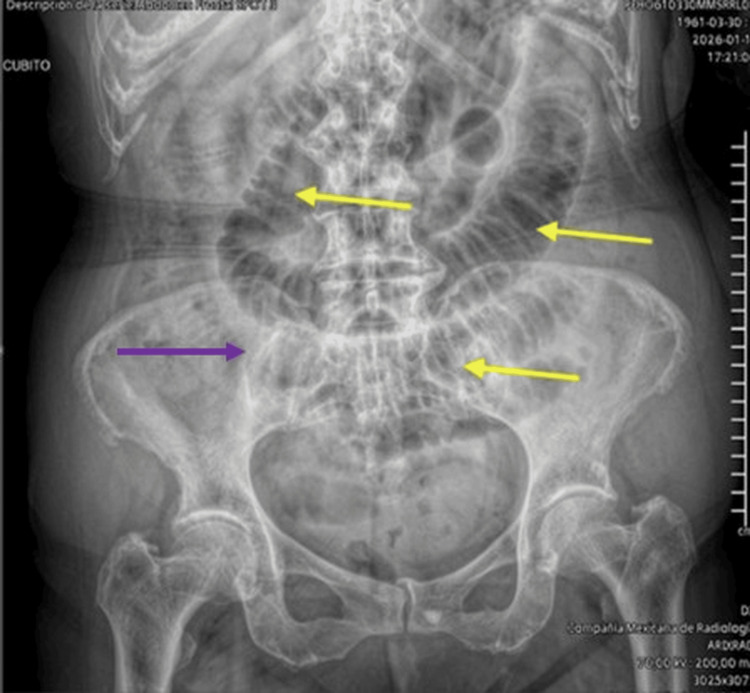
Supine X-ray showing dilated small bowel loops with a “stack of coins” appearance (yellow arrows) and a strangulated bowel loop within a Spigelian hernia (purple arrow), suggestive of mechanical small bowel obstruction.

Laboratory studies revealed leukocytosis with a left shift (Table 1). Upright and supine abdominal radiographs demonstrated dilated bowel loops with multiple air-fluid levels, the classic “coin-stack” sign, and a strangulated bowel loop within a Spigelian hernia representing the likely transition point and cause of the obstruction (Figures 1, 2).

**Table 1:** Laboratory findings on admission to the emergency department revealing marked leukocytosis with severe neutrophilia. Elevated serum creatinine levels were consistent with acute kidney injury.

Test	Result	Reference range
Glucose	70 mg/dL	70–99 mg/dL
Creatinine	1.6 mg/dL	0.6–1.2 mg/dL
Hemoglobin	14.6 g/dL	12.0–16.0 g/dL (female)
Hematocrit	44.4%	36–46% (female)
Platelets	442 × 10³/µL	150–450 × 10³/µL
White blood cells	24.49 × 10³/µL	4.0–10.0 × 10³/µL
Neutrophils	95.50%	40–70%

A diagnosis of hemodynamic instability and acute abdomen secondary to intestinal obstruction of undetermined etiology was established, and the patient was taken to the operating room for exploratory laparotomy via a midline supra- and infraumbilical incision.

**Figure 3:**
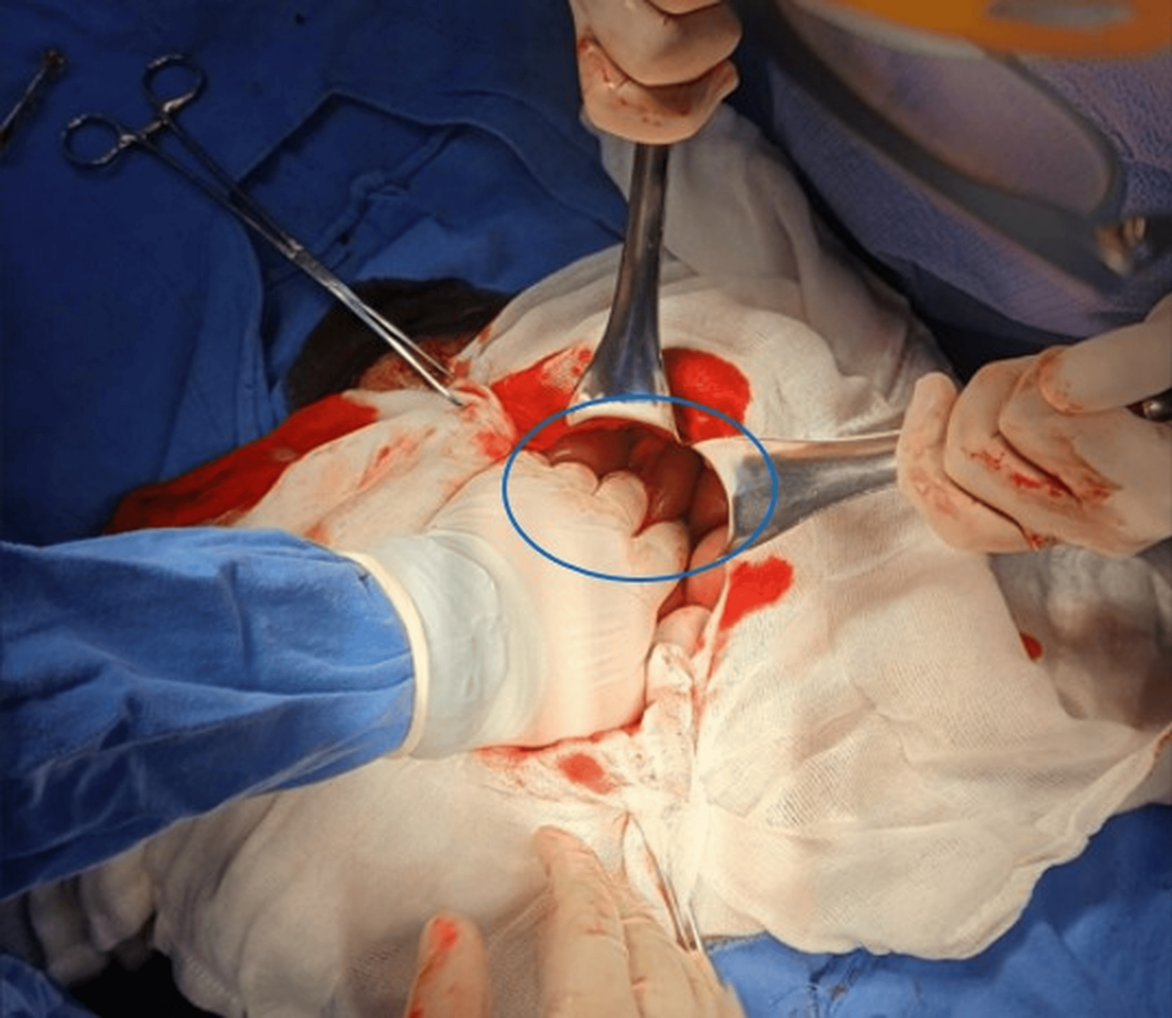
Intraoperative photograph showing distension of the small intestine (blue circle).

**Figure 4:**
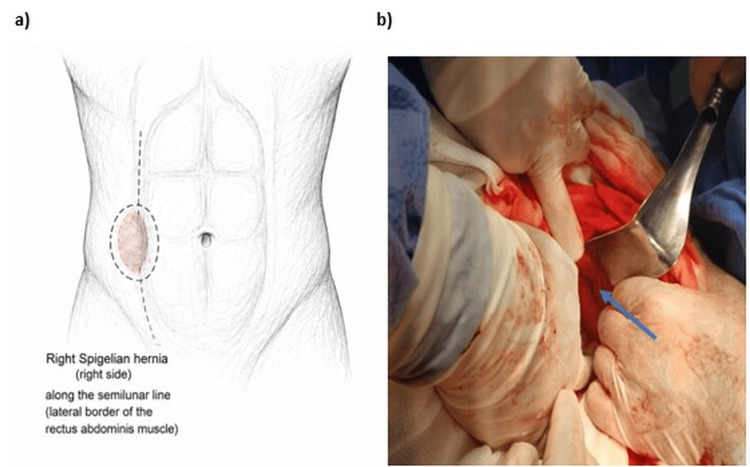
(a) Schematic illustration of a right Spigelian hernia located along the right semilunar line. (b) Intraoperative view from the intra-abdominal perspective through a midline laparotomy incision, demonstrating the Spigelian hernia defect along the right semilunar line (blue arrow).

**Figure 5:**
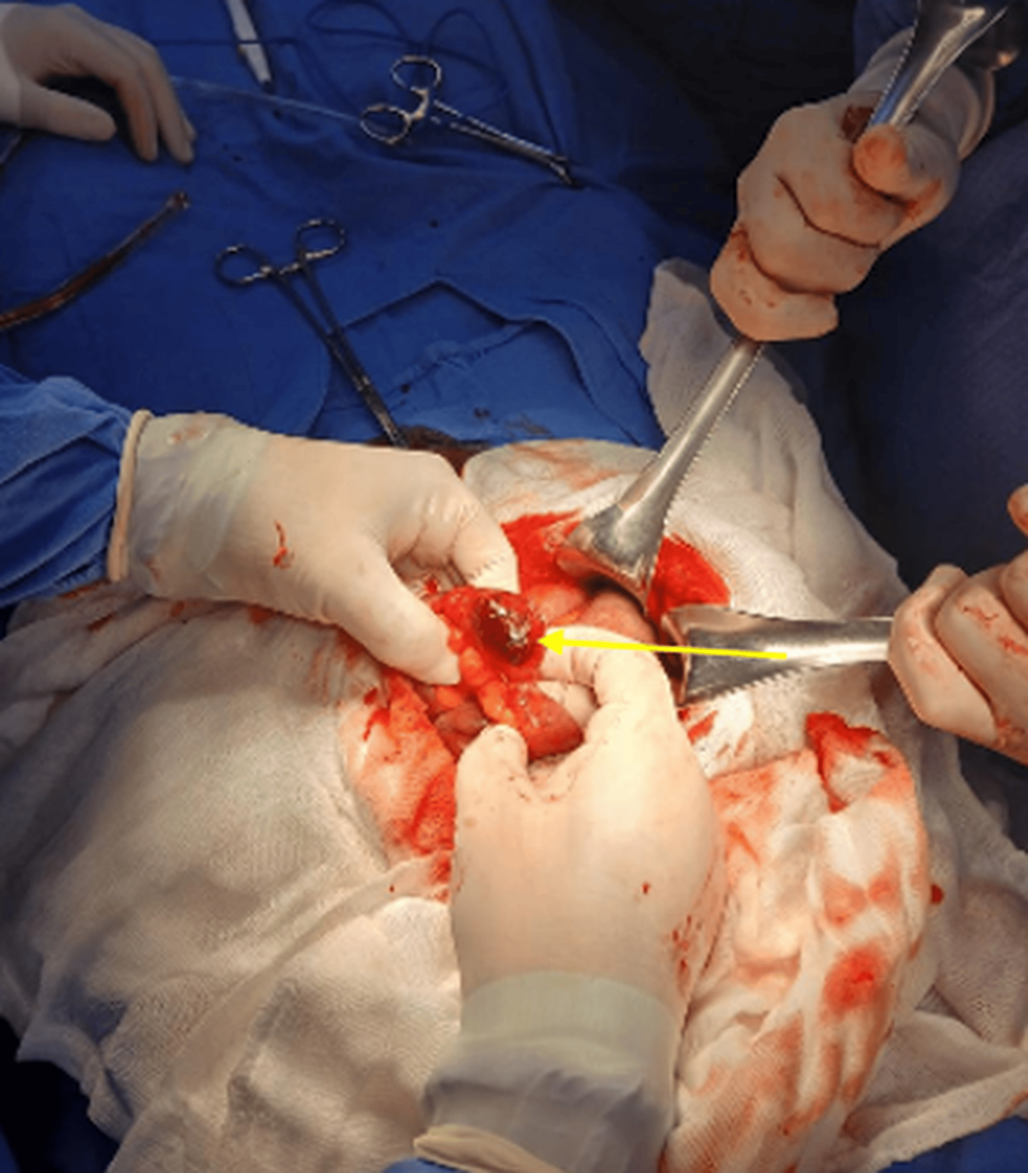
Intraoperative image showing a necrotic terminal ileal segment (yellow arrow).

Intraoperatively, a subaponeurotic mesh was identified at the umbilical level, along with markedly distended bowel loops (Figure 3). A Spigelian hernia defect measuring approximately 3 cm was found along the right semilunar line (Figure 4). The hernia sac, approximately 4 × 4 cm in size, contained a segment of terminal ileum with necrosis located 50 cm proximal to the ileocecal valve (Figure 5).

Approximately 10 cm of ileum were resected, followed by a two-layer end-to-end anastomosis. Primary closure of the hernia defect was then performed. A surgical drain was placed, and the abdominal wall was closed in layers.

The postoperative course was uneventful, with close clinical monitoring. By postoperative day five, pain was adequately controlled, with no evidence of anastomotic leak or fever. A liquid diet was initiated and well tolerated, with subsequent progression to a soft diet. Follow-up laboratory studies on postoperative day seven showed improvement in leukocytosis (Table 2). The patient was discharged from the hospital on postoperative day seven in stable condition.

**Table 2:** Laboratory results obtained seven days after surgery demonstrating resolution of leukocytosis and acute kidney injury, with normalization of neutrophil counts.

Test	Result	Normal range
Glucose	112 mg/dL	70–100 mg/dL
Creatinine	0.3 mg/dL	0.6–1.2 mg/dL
Hemoglobin	13 g/dL	12–16 g/dL (female)
Hematocrit	38.5%	36–46% (female)
Platelets	409 × 10³/µL	150–450 × 10³/µL
White blood cells	11.7 × 10³/µL	4.0–10.0 × 10³/µL
Neutrophils	69.60%	40–70%

## Discussion

Spigelian hernia is an uncommon abdominal wall defect, with an estimated incidence ranging from 0.1% to 2% of all abdominal hernias. Clinical diagnosis remains challenging due to its interparietal location between the layers of the abdominal wall, which often precludes identification of a palpable mass on physical examination. This anatomical characteristic contributes to delayed diagnosis, with a considerable proportion of cases presenting as surgical emergencies, most commonly due to incarceration or strangulation of hernia contents.

In the present case, the patient fit the epidemiological profile described in the literature, as she was a woman in her seventh decade of life with medical and surgical risk factors associated with weakening of the abdominal wall. Her clinical course, characterized by progressive abdominal pain, obstipation, and radiological findings consistent with intestinal obstruction, was highly suggestive of a mechanical obstructive process.

Intraoperative findings confirmed a Spigelian hernia with a defect measuring approximately 3 cm along the semilunar line, containing a necrotic segment of terminal ileum. This defect size is consistent with prior reports indicating that smaller fascial defects are associated with a higher risk of incarceration and vascular compromise [8]. The surgical management, consisting of resection of the necrotic bowel segment, end-to-end anastomosis, and primary repair of the hernia defect, is in accordance with current recommendations for complicated cases involving intestinal ischemia.

This case also highlights an important consideration in the emergency management of patients presenting with suspected intestinal obstruction and acute abdomen. Although computed tomography is often regarded as a valuable diagnostic tool for Spigelian hernia, clinical findings suggestive of acute abdomen, particularly when associated with hemodynamic instability and concern for bowel compromise, may warrant immediate surgical intervention without delaying treatment for additional imaging studies. In such scenarios, prolonging the preoperative period while awaiting advanced imaging may increase the risk of progression to more severe complications, including bowel perforation, worsening ischemia, sepsis, and increased morbidity. Therefore, in selected emergency settings, prompt operative exploration based on clinical judgment remains justified and may be critical to improving patient outcomes.

This case highlights the importance of considering this entity in the differential diagnosis of acute abdomen, particularly in patients with risk factors and signs of intestinal obstruction without an evident cause. It also underscores the critical role of timely surgical intervention as the definitive treatment to prevent potentially life-threatening complications.

## Conclusions

Spigelian hernia is a rare but clinically significant condition that should be considered in the differential diagnosis of acute abdomen, particularly in women in the fifth to seventh decades of life with risk factors for abdominal wall weakness. Its subtle clinical presentation and interparietal anatomical location frequently delay diagnosis, thereby increasing the risk of incarceration and strangulation. Early recognition, supported by appropriate imaging when clinically feasible and without delaying urgent surgical management, is essential to reduce morbidity and prevent life-threatening complications. This case highlights the importance of maintaining a high index of suspicion and prioritizing timely surgical intervention in patients presenting with unexplained intestinal obstruction, particularly when bowel compromise is suspected.
